# Audit of Radiology Request Form for Completion and Usefulness of Clinical History: Teaching Hospital Experience, Ghana

**DOI:** 10.1155/2021/5583442

**Published:** 2021-05-12

**Authors:** Bashiru Babatunde Jimah

**Affiliations:** Department of Medical Imaging, College of Health Sciences, University of Cape Coast, Cape Coast, Ghana

## Abstract

**Background:**

The role of the “traditional” radiologist has shifted from imaging centered to patient focus, which underscores the utmost importance of the clinical radiologist in the multidisciplinary team in patient management. For the clinical radiologist to effectively play this key role, the referring clinician must provide adequate and useful patient information to assist the radiologist in making a diagnosis or provide differential diagnosis. The objectives were to assess the level of completion of the radiology request form and to determine whether the clinical history provided aided in the final impression/diagnosis. *Materials and Method*. We conducted a prospective review of 500 radiology request forms at the Cape Coast Teaching Hospital (CCTH) between September and October 2018. The forms were consecutively sampled and reviewed for each field/area such as patient's name, age, and clinical history. Data were analyzed descriptively for the level of completeness and usefulness of clinical history entered by the clinician.

**Results:**

No request form was completed in full. All the request forms did not have X-ray serial number and previous examination details documented. The proportions of forms with various fields completed were as follows: more than 90% of the forms had patient's name, investigation required, date of the request, doctor's name, and clinical history fields filled. The patient's age, patient's ward/address, and doctor's address were filled in 88%, 75%, and 18.4%, respectively. Twenty percent of the request forms were not useful to the radiologist in the final diagnosis.

**Conclusion:**

A significant proportion of radiology request forms are incompletely filled and therefore denies the radiologist, the critical information needed to make a diagnosis, or narrow differential diagnosis.

## 1. Introduction

The concept of “Lean Thinking in Healthcare” is an adoption from the auto and manufacturing industry which seeks to improve workflow, efficiency, and customer satisfaction [1]. This concept has been used in the United States and United Kingdom to improve the efficiency of pathology and emergency departments [1]. The concept, if implemented, can improve patient satisfaction by removing waste in the radiology department, from request form writing to delivery of reports to patients. The entire principle of the concept of “Lean Thinking” in the radiology department is beyond the scope of the current write up and will be discussed extensively in the upcoming paper “*The concept of Lean Thinking in Radiology, what must change in Ghana*.” This paper focuses on the completion of the various aspects of the request form and ways to improve the form.

The management of patient requires a multidisciplinary approach involving the radiologist and the clinician. Utmost importance of the clinical radiologist is the reflection of the evolving role of the “traditional” radiologist from an imaging centered to patient focus [2]. The radiologist role is to aid other colleagues in reaching their diagnosis and, since the advent of interventional radiology, provide treatment for various conditions. For the clinical radiologist to function effectively, it is imperative that the referring clinician provides adequately filled requisition forms.

The radiology request forms are clinical and legal documents, completed by a referring clinician or his/her surrogate, and serve to communicate the procedure required and the reasons for the procedure [3]. The guidelines of the Royal College of Radiologist (RCR) suggest that this request form should be completed adequately and legibly to avoid any misinterpretation [4]. A comparison of the American College of Radiology and the Royal College of Radiology shows that a radiology request form should contain the following information [5, 6]: the clinical background; the question to be answered; the patient's name, age, address, and telephone number; the ward; the name and signature of the requesting doctor; the name of the consultant responsible for the patient's well-being; and the date.

The forms provide the clinical question to be answered by the radiologist. Some diseases have similar radiographic patterns, and their elucidation requires adequate patient information. An incompletely filled form will make it difficult for the radiologist to narrow the differential diagnosis for certain imaging patterns. It may also lead to unnecessary investigations, increased hospital stay, repeated exposure to radiation, and delayed management of the patient with increased cost to the patient and the hospital.

The aim of this study is to assess the level of completion of the radiology request form and whether the clinical information provided aided in the final impression by the radiologist. This study did not assess the appropriateness of the request form.

## 2. Materials and Methods

A prospective review of the radiology request was conducted at the Cape Coast Teaching Hospital (CCTH) between September and October 2018. The Cape Coast Teaching Hospital is located within the Cape Coast Metropolis in the central region of Ghana. It is a 234-bed facility and serves as a tertiary referral center for the central and western regions. It serves as a teaching hospital for the University of Cape Coast School of Medical Sciences and Allied Health Science faculty. It is a residency training center for the Ghana College of Physicians and Surgeons.

Prior to the study, ethical approval was sought from the Ethical Review Committee of the Cape Coast Teaching Hospital.

The CCTH Department of Radiology receives paper request forms for various imaging modalities. The request forms are presented by the patients on the day of their investigation. A total of 500 readily available request forms were analyzed within one-month period of the study from inpatient and outpatient clinics of the CCTH, private hospitals, and public hospitals within central and western regions. The modalities included plain radiographs for radiologist's report (35), computed tomography scan (77), general ultrasonography: abdomen, pelvis, and small parts (300), vascular ultrasound scan (35), hysterosalpingography (21), mammography (24), and special studies (8) (barium enema, barium swallow, barium meal, MCUG, RUG, and distal loopogram).

We assessed each request form for the filling of all the fields on the form (demographics of patients/patient identifiers (age, name, and address), history/clinical information, investigation requested, demographics of the referring physician (name and address), X-ray serial number, previous serial number/previous exam details, and date of the request. The audit did not assess the appropriateness of the investigation requested.

Data were analyzed descriptively using percentages. The clinical history was considered “useful” if it contributed to the final impression/diagnosis and “not useful” if it did not contribute to the final impression/diagnosis.

## 3. Results

**Figure 1:**
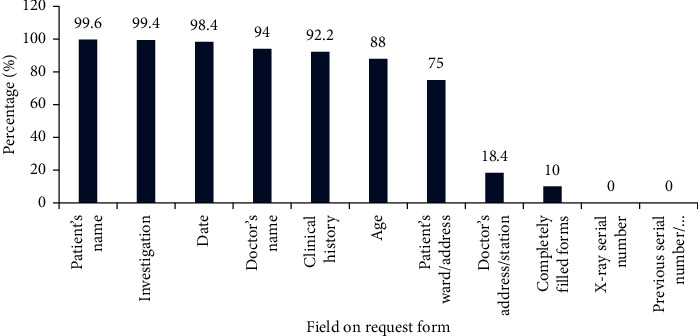
Proportion of forms with fields completely field.

A total of 500 request forms were analyzed in the study. None of the forms were completely filled. Details of form completion are as shown in Figure 1. Almost all the request forms had the patients name filled (99.6%) except two (0.4%). The investigation requested was filled in 99.4%, and only three were not filled. The date and doctor's name were filled in 98.4% and 94%, respectively. The other fields such as the clinical history, age, patient's ward/address, and doctor's address/station were completed, respectively, in 92.2%, 88%, 75%, and 18.4%. X-ray serial number and previous serial number/previous exam details were not filled in any of the forms.

**Figure 2:**
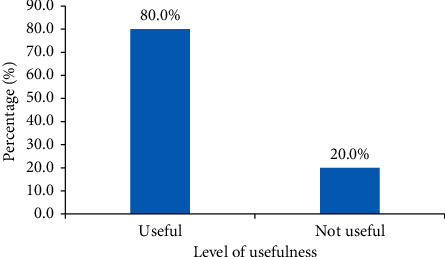
Usefulness of clinical information provided.

Figure 2 shows the level of usefulness of clinical information of request forms. Twenty percent (100/500) of the request forms (includes request forms with no clinical history and request forms with unhelpful clinical information) were not useful, which implies that they did not contribute to the final diagnosis or impression.

**Figure 3:**
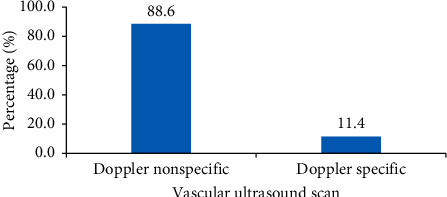
Category of request for vascular ultrasound.

A total of 35 vascular ultrasound scan were requested which included peripheral venous and arterial studies. 31 (88.5%) of the requests were nonspecific (venous or arterial or both), and only 4 were specific for venous Doppler (Figure 3).

Fifty percent (250/500) of the request forms had abbreviations that did not suit the internationally recognized, particularly in areas such as the age, patient's ward/address, clinical history, and investigation (Table 1).

## 4. Discussion

Management of the patient requires a multidisciplinary approach that is based on adequate communication between the various team members, in order to provide the patient with the best possible service [2–4]. The radiology request form is an important communication tool between the managing clinician and the radiologist [3, 4, 6]. At CCTH, multidisciplinary board meetings and interdepartmental clinical meetings are organized routinely to review clinical activities within a period. The radiology requisition form has been an item often discussed to increase awareness of the importance of the information provided on the request form and to sensitize referring clinicians to adequately fill the form to aid the radiologist to narrow the differential diagnosis for certain imaging patterns.

Most studies show that there is a global deficiency in the completion of the radiology requisition form [2, 3, 6–9]. The study findings showed that none of the request form was completely filled. This is consistent with the findings by Akinola et al. [6], and Depasquale and Crockford [2] found 4% of request forms fully filled. Seventy-five percent (*n* = 375) had patient's ward/address fully filled in this study, similar to the findings by Depasquale and Crockford [2] who found 77% of request form had addresses in their study, contrary to Akinola et al. [6] who found only 2.1% of request form with addresses. For most inpatients, the addresses will help the imaging department submit the report to reduce waiting time.

The findings showed that 99.6% of the forms had the names of the patients adequately filled. Similar observation was made by Agi et al. [8] who reported that all the forms reviewed had the names of the patients properly filled. Patient identifiers such as name are of utmost importance and must be included at all times.

It was surprising to see that 3 (0.6%) of the request form did not have any stated investigation to be performed, contrary to Agi et al. [8] who had 100% of the investigated stated. The radiologist had to take a clinical history from the patient and decide on the appropriate procedure. Such omissions may increase the waiting time, increase cost of transportation, and ultimately patient dissatisfaction.

The doctor's name and doctor's ward/address were found in 94% and 18.4%, respectively. In the study done by Akintomide et al. [7], 86% of the forms had medical officer's name provided and 24.4% of the medical officers provided their names in Akinola et al.'s [6] study. We observed that the doctors who provided their ward/address (18.4%) were predominantly on National Health Insurance request forms which makes it mandatory in others for claims to be paid. Occasionally, radiologists seek clarification on the information provided. The absence of doctor's information may deny the radiologist the needed patient information. This may potentially increase the reporting time or provide a wide range of differential diagnosis which may not help in the management of the patient.

**Figure 4:**
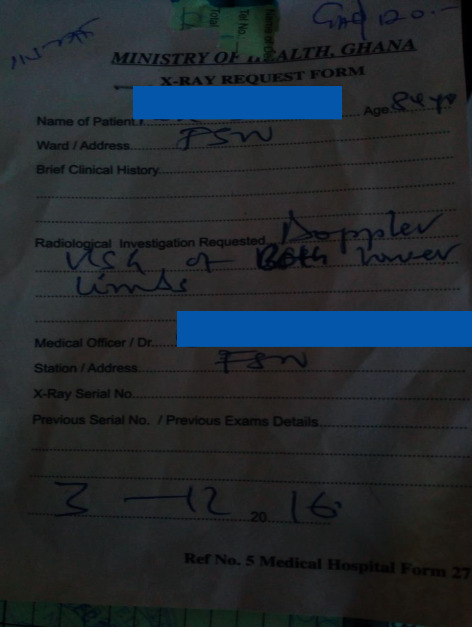
A sample of a request form. No clinical history is provided. The investigation requested is not specific as it has cost implications to the patient. The X-ray serial number and previous examination details were not provided. Unknown medical abbreviation is noted at ward/address.

Arterial or venous disease often has specific presenting complaint and must be stated. Most health facilities do not accept the National Health Insurance for Doppler investigations, and most patients do not have private insurance. Clients therefore have to pay out-of-pocket for these investigations. Cost of performing both arterial and venous studies as stated in Figure 4 is likely in the range of 1000–1400 Ghana Cedis. For only venous studies, the cost is likely 400–600 Ghana Cedis or 600–800 Ghana Cedis for arterial studies. It is therefore important to refer clinician's state clearly what is required to avoid unnecessary cost to the patient.

The result shows that 13.2% of the clinical information provided was not useful in arriving at the final impression, and 6.8% of the request forms analyzed did not provide any clinical information at all. This means that 20% of the request forms were not useful to the radiologist. Akinola et al. [6] found that request forms that provided adequate clinical history received radiologist reports that were more helpful in the management of the patients. This study shows that 80% of the request forms received had useful clinical information. This could be due to the prior in-service training to the various departments about the need to adequately fill the request forms. This finding is compared to the 100% clinical history found by Akinola et al. [6] and 71% by Akintomide et al. [7].

The radiology request form does not include the clinical question to be answered by the radiologist, and therefore, the radiologist must deduce the question from the clinical information provided by the clinician. The clinical question to be answered and the clinical information provided are important for the radiologist to decide whether the investigation requested will answer the question asked, to avoid exposing the patient to unnecessary radiation, decide on the protocol for the study, and aid in the final diagnosis or differential diagnoses [2, 6, 10]. Periodic education of referring clinicians is imperative to ram home this message.

Our review further showed that more than 50% of the request forms had abbreviations that are not internationally recognized. Fields with such abbreviations included the age, ward's/patient's address, clinical history, and investigation. Typical example is FMW, in our institution, which refers to female surgical ward but internationally represents formulated molecular weight [11]. Other internationally recognized abbreviations are OPD (outpatient department), AKI (acute kidney injury), HCC (hepatocellular carcinoma), CKD (chronic kidney disease), TIA (transient ischemic attack), CVA (cerebrovascular accident), RIF (right iliac fossa), LT (left), RT (right), ? (query/question), # (fracture), CXR (chest radiograph), USG (ultrasonography), CT (computed tomography), mammo (mammography), HSG (hysterosalpingography), ICSOL (intracranial space occupying lesion), Paeds (paediatrics), and RUG (retrograde urethrogram) [11].

Our review showed that all the referring doctors failed to include their contacts to allow the radiologist to probe for further information. This observation in practice is contrary to the guidelines proposed by the Royal College of radiology calling on referring clinician to provide the contact details [4, 5]. In our experience at our institution, referring clinicians are contacted through the hospital or regional platforms. Despite the perceived challenges, effective communication is necessary between radiologist and referring clinicians, and it must be a concern for a multidisciplinary management team.

## 5. Conclusion

A significant proportion of radiology request forms are incompletely filled and therefore denies the radiologist, the critical information needed to make a diagnosis or narrow differential diagnosis. Attitudinal change among referring clinicians in filling radiology request form will be essential in effective diagnosis and management of patients.

### 5.1. Recommendation

In line with the study findings and discussions on the possible actions or changes that could be implemented in order to reach this goal, we recommend the following:

- The Ministry of Health/Ghana Health Service should review the current radiology request form to suit international best practice and ensure its adoption by all health facilities (both public and private)
- The X-ray serial number and previous serial number/previous exam details must be removed from the current request form and replaced with findings of previous exams if any
- The transition from the paper-based to the paperless system in most hospitals, including Cape Coast Teaching Hospital, should ensure that electronic request forms meet the international best practice
- All electronic request forms should ensure that all aspects of the forms are filled before it can be transmitted to the imaging department
- Frontline staff at the imaging department should be trained to return any inadequately completed forms to referring clinician who still uses the paper-based forms
- Completion of the request form should be part of the orientation for new doctors and periodic in-service training for all clinicians and physician assistants

## Figures and Tables

**Table 1:** Abbreviations on request forms that are not internationally recognized, CCTH.

Abbreviation	Referring clinician meaning	Internationally accepted meaning
A	Adult	Adenine, adenosine, alanine, ampere/amp, artery, vitamin A
MMW	Male medical ward	Medium molecular weight
FMW	Female medical ward	Formulated molecular weight
FSW	Female surgical ward	No medical meaning
CLD	Chronic liver disease	Chronic lung disease, cholestatic liver disease
HPT	Hypertension	Home pregnancy test
LAP	Lower abdominal pain	Left atrial pressures, laparoscopy/laparoscopic

## Data Availability

The data used to support the findings of this study are included within the article.
